# Dose-dependent IFN programs in myeloid cells after mRNA and adenovirus COVID-19 vaccination

**DOI:** 10.1172/jci.insight.199245

**Published:** 2026-02-23

**Authors:** Giray Eryilmaz, Yilmaz Yucehan Yazici, Radu Marches, Eleni P. Mimitou, Lisa Kenyon-Pesce, Kim Handrejk, Sonia Jangra, Michael Schotsaert, Adolfo García-Sastre, George A. Kuchel, Jacques Banchereau, Duygu Ucar

**Affiliations:** 1 The Jackson Laboratory for Genomic Medicine, Farmington, Connecticut, USA.; 2 Immunai, New York, New York, USA.; 3 UConn Center on Aging, UConn Health, Farmington, Connecticut, USA.; 4 Department of Microbiology,; 5 Global Health and Emerging Pathogens Institute,; 6 Marc and Jennifer Lipschultz Precision Immunology Institute,; 7 Icahn Genomics Institute,; 8 Department of Medicine, Division of Infectious Diseases,; 9 The Tisch Cancer Center, and; 10 Department of Pathology, Molecular and Cell-Based Medicine, Icahn School of Medicine at Mount Sinai, New York, New York, USA.; 11 Immunoledge LLC, Montclair, New Jersey, USA.; 12 Department of Genetics and Genome Sciences, University of Connecticut Health Center, Farmington, Connecticut, USA.

**Keywords:** Immunology, Public Health, COVID-19, Innate immunity, Vaccines

## Abstract

**BACKGROUND:**

The SARS-CoV-2 pandemic provided a rare opportunity to study how human immune responses develop to a novel viral antigen delivered through different vaccine platforms. However, to date, no study has directly compared immune responses to all 3 FDA-approved COVID-19 vaccines at single-cell multiomic resolution.

**METHODS:**

We longitudinally profiled SARS-CoV-2–naive adults (*n* = 31) vaccinated with BNT162b2, mRNA-1273, or Ad26.COV2.S, integrating plasma cytokines, antibody titers, and single-cell multiomic data (DOGMA-Seq).

**RESULTS:**

We discovered a distinct, transient IFN program termed ISG-dim, which emerged specifically 1–2 days after the first mRNA dose in approximately 10% of myeloid cells. This state was characterized by ISGF3 complex activation and its target genes (e.g., *MX1*, *MX2*, *DDX58*), with transcriptional and epigenetic profiles distinct from the robust IFN program observed after mRNA boosting or a single Ad26.COV2.S dose (ISG-high). In vitro stimulation of human monocytes showed that IFN-α alone recapitulates ISG-dim, whereas both IFN-α and IFN-γ are required for ISG-high.

**CONCLUSION:**

These findings define dose-dependent IFN programming in human myeloid cells and highlight mechanistic differences between priming and boosting, with implications for optimizing vaccine platform choice, dose scheduling, and formulation.

**FUNDING:**

NIH grants AI142086, U19 AI135972, U01 AI165452, U01 AI165452, R01 AI160706, and P30 AG067988.

## Abstract 2

A high-resolution multi-omics study reveals that the primary and secondary mRNA vaccines elicit distinct innate responses driven by Type-I and Type-II interferons, respectively.

## Introduction

The SARS-CoV-2 pandemic posed unprecedented global health challenges but also created a rare scientific opportunity to study how human immune responses develop to a novel viral antigen introduced through different vaccine platforms. Among these, the novel and highly effective mRNA vaccines BNT162b2 (Pfizer-BioNTech) and mRNA-1273 (Moderna) utilized lipid nanoparticles to deliver nucleoside-modified RNA encoding the SARS-CoV-2 spike protein (1–4). An alternative platform, Ad26.COV2.S (Johnson & Johnson), delivered spike-encoding DNA via a nonreplicating adenoviral vector (5). Although all 3 vaccines target the same antigen, their distinct formulations and platforms provide a unique framework to dissect human immune priming and boosting. Despite this opportunity, to our knowledge, no prior study has performed a head-to-head, single-cell multiomic comparison of all 3 FDA-approved COVID-19 vaccines in SARS-CoV-2–naive individuals.

Immune responses, particularly adaptive immune responses following the second dose of mRNA vaccines, have been well characterized, including the robust expansion of CD4^+^ and CD8^+^ T cells, memory B cells, and the production of SARS-CoV-2–neutralizing antibodies (6–15). In contrast, early innate responses, especially in human myeloid cells after the first dose, remain poorly understood. Although some studies noted IFN-stimulated gene (ISG) induction (13, 16–18), the magnitude, timing, and cell-type specificity of this response have not been resolved. Our study fills this critical gap by providing a single-cell, multiomic map of early innate priming in humans after mRNA vaccination.

Here, we leveraged the unique conditions of the early days of the pandemic to study how immune responses develop in SARS-CoV-2–naive healthy adults (*n* = 31) vaccinated with either mRNA (BNT162b2) (3), mRNA-1273 (4), or the adenoviral vector (Ad26.COV2.S) (5) COVID-19 vaccines. Using single-cell multiomics alongside cytokine and antibody profiling, we mapped the temporal dynamics of innate responses at single-cell resolution. We uncovered a distinct myeloid priming program induced by type I IFNs after the first mRNA dose, and a broader boosting response involving both type I and type II IFNs after the second mRNA dose or Ad26.COV2.S. By defining dose-specific and vaccine platform–specific IFN programs in human myeloid cells, our study provides a mechanistic framework for optimizing vaccine platform choice, scheduling, and formulation, with implications for improving protection in vulnerable populations.

## Results

### Head-to-head comparison of 3 COVID-19 vaccines in SARS-CoV-2–naive adults.

**Figure 1:**
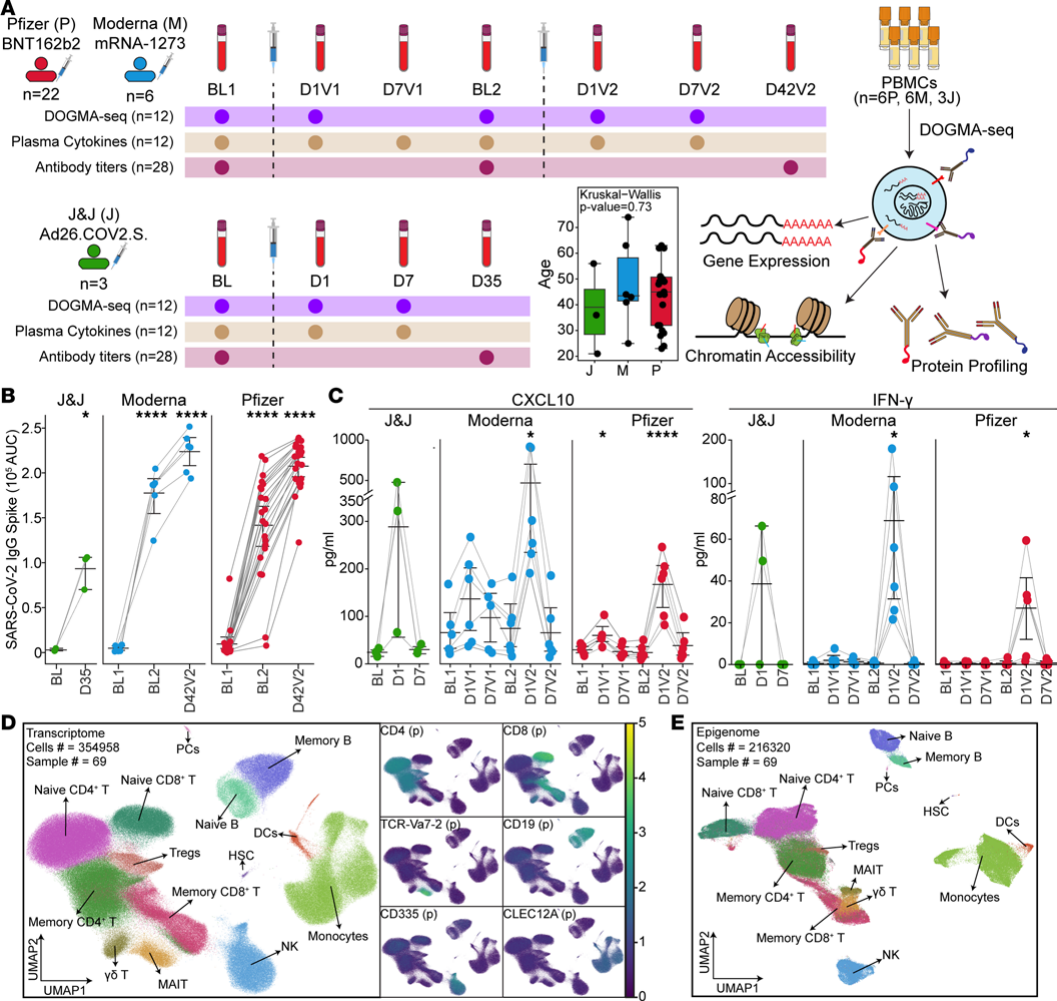
A longitudinal systems immunology study of immune responses to COVID-19 vaccines. (**A**) Study design. Blood samples were collected longitudinally from individuals (*n* = 31) vaccinated with either 2 doses of mRNA vaccines (Pfizer-BioNTech or Moderna) or 1 dose of the adenovirus-based Johnson & Johnson vaccine. Antibody titers were measured before and 3–4 weeks after each vaccination. A subset of donors (6 Pfizer, 6 Moderna, 3 Johnson & Johnson) was selected for in-depth profiling, including serum cytokine analysis and DOGMA-Seq. Age distributions among vaccine groups were compared using the Kruskal-Wallis test. (**B**) IgG titers against SARS-CoV-2 spike protein measured by ELISA before and after vaccination. Antibody titers further increased after the mRNA booster dose. (**C**) Serum levels of CXCL10 and IFN-γ at key time points were quantified using ELLA. Both cytokines increased on day 1 (D1) after the adenoviral vaccine and following the mRNA booster dose. (**D** and **E**) UMAP projections of DOGMA-Seq data for transcriptome (**D**, scRNA-Seq) and chromatin accessibility (**E**, scATAC-Seq) modalities. Canonical lineage markers (surface protein and gene expression) annotated major immune cell populations. For **B** and **C**, statistical significance was assessed using 2-tailed paired *t* tests: *P < 0.05, ****P < 0.0001.

Thirty-one healthy adults with no prior history of SARS-CoV-2 infection or vaccination were enrolled between spring and summer 2021 at the UConn Health Center. Of these, 28 participants received mRNA vaccines, including both BNT162b2 (*n* = 22) and mRNA-1273 (*n* = 6), and 3 received the adenoviral vector vaccine Ad26.COV2.S (Figure 1A and Supplemental Table 1; supplemental material available online with this article; https://doi.org/10.1172/jci.insight.199245DS1). Age distributions were comparable across groups (Figure 1A and Supplemental Table 1).

mRNA vaccines were administered in 2 doses, whereas Ad26.COV2.S was administered as a single dose, according to CDC recommendations. Blood samples were collected at key time points: baseline (prevaccination), day 1 (innate responses), day 7 (early adaptive responses), and days 25–35 (antibody responses) after each vaccine dose (Figure 1A and Supplemental Table 1), totaling 7 time points for mRNA vaccines and 4 time points for Ad26.COV2.S. The isolated PBMCs were used for genomic profiling, and plasma samples were used for cytokine and antibody titer profiling.

Anti–SARS-CoV-2 spike protein IgG titers were measured by ELISA at baseline and 3–4 weeks after each vaccination. All vaccine groups showed a significant increase in antibody titers after the first dose (*P* < 0.0001), followed by an additional increase after the second mRNA dose (Figure 1B). After 2 doses, individuals vaccinated with mRNA vaccines had significantly higher titers (*P* = 0.0369) than those receiving a single dose of Ad26.COV2.S (Supplemental Figure 1A). Despite the dose difference between BNT162b2 (30 μg) and mRNA-1273 (100 μg), antibody titers were comparable between the 2 mRNA groups at both time points (Supplemental Figure 1A). No correlation was observed between antibody titer levels and participant age (Supplemental Figure 1B).

Plasma levels of 6 cytokines were quantified at baseline, day 1, and day 7 after each vaccination using the ELLA platform (Bio-Techne) (Figure 1C, Supplemental Figure 1C, and Supplemental Table 2). The first dose of mRNA vaccines modestly elevated CXCL10 levels at day 1 (*P* = 0.14 for Moderna, *P* = 0.013 for Pfizer), and the second dose induced stronger induction of both CXCL10 and IFN-γ (CXCL10: *P* = 0.019 for Moderna, *P* = 0.00036 for Pfizer; IFN-γ: *P* = 0.019 for Moderna, *P* = 0.0125 for Pfizer, Figure 1C). In contrast, both IFN-γ and CXCL10 levels increased to levels comparable to those induced by mRNA vaccines at day 1 after Ad26.COV2.S. Furthermore, we observed a correlation between IFN-γ levels after a booster vaccine (day 1, vaccine dose 2) and fold change of IgG titers (day 42 vs. baseline), suggesting a potential link between IFN responses and humoral immunity (Supplemental Figure 1D). No significant changes were observed for IL-8, TNF-α, CCL2, or IL-10 levels after any vaccine (Supplemental Figure 1C).

Together, these data showed robust antibody responses and limited cytokine induction after the first mRNA dose, followed by enhanced humoral and innate cytokine responses after the second dose. No significant differences were observed between the 2 mRNA platforms despite significant differences in their doses.

### Longitudinal profiling of PBMCs with a single-cell multiome assay.

To investigate the cellular dynamics underlying vaccine-induced immune responses, we performed longitudinal profiling of PBMCs from 15 participants who received mRNA-1273 (*n* = 6), BNT162b2 (*n* = 6), or Ad26.COV2.S (*n* = 3) (Figure 1A) using DNA-Oriented Genome and Transcriptome Mapping by Analysis sequencing (DOGMA-Seq). DOGMA-Seq is a multimodal single-cell assay that enables the simultaneous measurement of surface protein expression, gene expression, and chromatin accessibility from the same cells (19). DOGMA-Seq libraries were generated at baseline, day 1, and day 7 for Ad26.COV2.S; at baseline and day 1 after the first mRNA vaccine dose; and at baseline, day 1, and day 7 after the second mRNA dose (Figure 1A). A total of 69 samples were multiplexed using Cell Hashing (20) and sequenced in batches of 6–8.

After quality control and filtering, we obtained 354,958 high-quality cells for single-cell RNA-Seq (scRNA-Seq) and 216,320 for single-cell assay for transposase-accessible chromatin sequencing (scATAC-Seq) (Figure 1, D and E, Supplemental Figure 1E, Supplemental Figure 2, Supplemental Figure 3, A and B, and Supplemental Table 3). We recovered approximately 5,000 high-quality cells per sample (donor and time point). Surface protein expression and canonical marker genes identified major immune cell populations including monocytes, NK cells (CD335), B cells (CD19), CD4^+^ T cells (CD4), CD8^+^ T cells (CD8), γδ T cells (TCR-Vδ2), and mucosal-associated invariant T (MAIT) cells (TCR-Vα7.2) (Figure 1D and Supplemental Figure 1E). Cell-type annotations from the RNA-Seq modality were transferred to the ATAC-Seq modality (Figure 1E). This dataset enabled a high-resolution, longitudinal analysis of vaccine-induced immune responses, with transcriptional and epigenetic programs captured from the same cells providing direct insights into coordinated regulatory states at single-cell resolution for each vaccine.

### Strong transcriptional IFN responses in myeloid cells after the second mRNA and Ad26.COV2.S vaccines.

**Figure 2:**
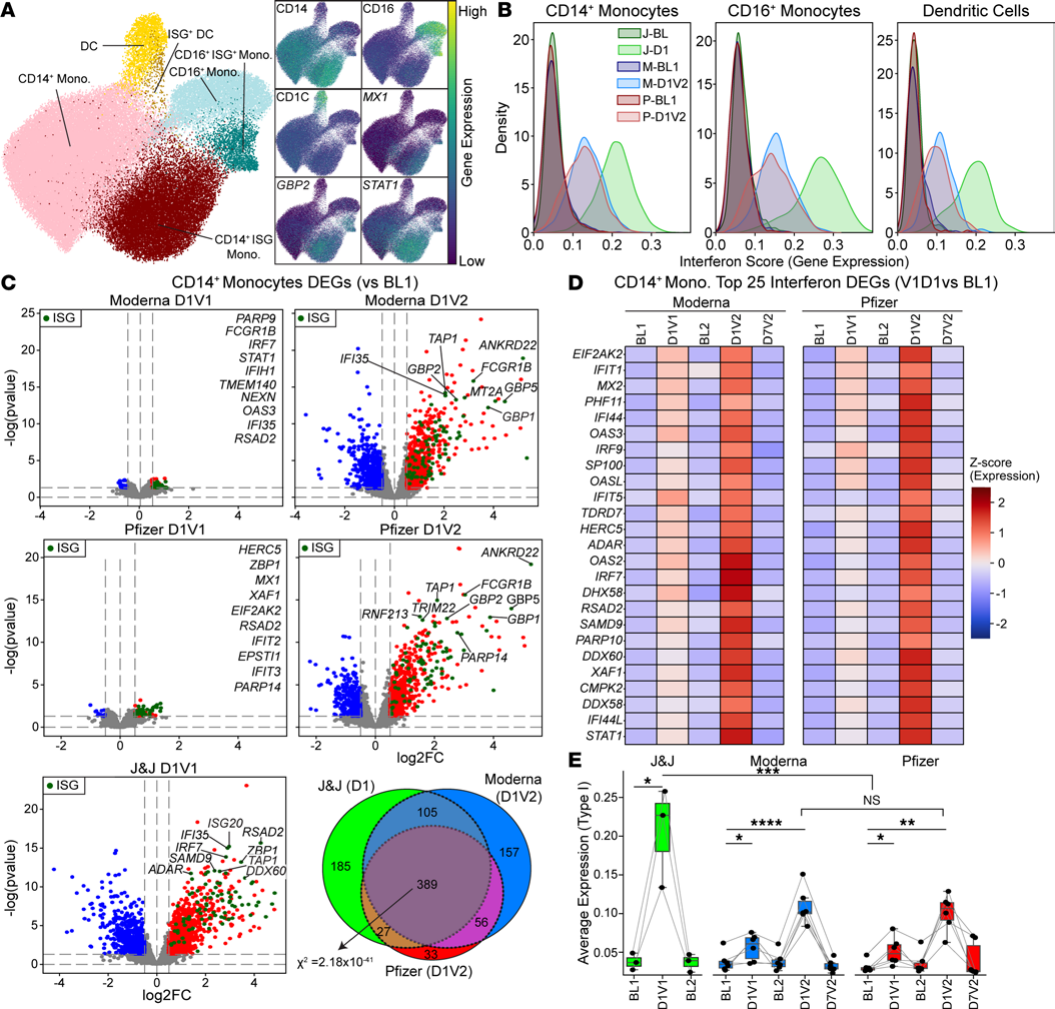
mRNA vaccines induce a robust IFN response after the booster dose. (**A**) UMAP representation of the myeloid compartment from DOGMA-Seq, annotated using canonical markers to identify CD14^+^ monocytes, CD16^+^ monocytes, and DCs. IFN-stimulated gene (ISG) subsets were defined based on the expression of ISG markers in each subset. (**B**) Kernel density estimation plots of ISG scores for each vaccine and time point show that Johnson & Johnson elicits a strong IFN response 1 day after vaccination; mRNA vaccines induce a robust response after the booster. (**C**) Differential gene expression analysis in CD14^+^ monocytes comparing postvaccination time points (D1V1, D1V2) to baseline. Thresholds for significance: log_2_FC > 0.5 and *P* < 0.05. ISGs are highlighted in green. Venn diagram shows the overlap of differentially expressed genes (DEGs) from D1 (Johnson & Johnson), D1V2 (Moderna), and D1V2 (Pfizer). A χ^2^ test was conducted across the 3 groups to evaluate whether the overlap of DEGs was statistically significant, indicating a shared transcriptional response. (**D**) Heatmap showing the top 25 IFN-related genes upregulated 1 day after the booster (D1V2) for mRNA vaccine recipients. (**E**) Type-I IFN expression score calculated from manually curated list (*n* = 43) shows stronger type I IFN response after Johnson & Johnson vaccination compared with mRNA responses. Statistical significance between time points was assessed using a 2-tailed paired *t* test; comparisons between adenovirus vaccine and mRNA vaccine (Moderna and Pfizer combined) groups were performed using the unpaired *t* test: NS, nonsignificant, *P < 0.05, **P < 0.01, ***P < 0.001, ****P < 0.0001.

Clustering of single-cell transcriptomic profiles from the myeloid compartment (*n* = 71,184 cells) revealed 3 major subsets: CD14^+^ monocytes, CD16^+^ monocytes, and DCs (Figure 2A and Supplemental Figure 4A). We obtained a median of 628 CD14^+^ monocytes, 112 CD16^+^ monocytes, and 40 DCs per sample (donor and time point) (Supplemental Figure 4B). One day after Ad26.COV2.S vaccination, the frequency of CD14^+^ monocytes increased substantially, comprising up to 50% of total PBMCs on average, a 2.7-fold expansion relative to baseline (Supplemental Figure 4C). No significant changes in cell composition were observed for CD16^+^ monocytes and DCs. In contrast, mRNA vaccination elicited a more modest but statistically significant increase in CD14^+^ monocytes after both the first dose (*P* = 0.002, fold change = 1.25) and the second dose (*P* = 0.0034, fold change = 1.72) (Supplemental Figure 4C).

Within each myeloid subset, we detected ISG^+^ cells characterized by the high expression of ISGs, including critical antiviral and signaling molecules *MX1*, *GBP2*, *WARS*, and *STAT1 (*Figure 2A and Supplemental Figure 4A). These ISG^+^ populations significantly expanded at day 1 after Ad26.COV2.S and after the mRNA second dose (Supplemental Figure 4D), as confirmed by cumulative ISG scores calculated at the single-cell level using previously published gene set modules (*n* = 96 genes) (21) (Figure 2B). Notably, this IFN response was stronger after Ad26.COV2.S compared with the mRNA second dose (*P* = 0.004).

To further quantify transcriptional changes upon vaccination responses, we performed differential expression analysis by comparing postvaccination time points to baseline in CD14^+^ and CD16^+^ monocytes and DCs (Figure 2C and Supplemental Table 4). The highest number of differentially expressed genes was detected at day 1 after Ad26.COV2.S and at day 1 after the second mRNA vaccination in CD14^+^ monocytes (Figure 2C). Across all platforms, 389 genes were commonly induced (χ^2^ test, *P* = 3.66 × 10^–19^) (Figure 2C), and these were enriched in IFN-associated pathways (Supplemental Figure 4E and Supplemental Table 4), suggesting a shared core IFN response in CD14^+^ monocytes upon mRNA booster vaccination and after Ad26.COV2.S.

Although relatively few differentially expressed genes were detected after the first mRNA vaccination in CD14^+^ monocytes, several ISGs, including *OAS3, IFI44,* and *MX2*, were upregulated (Figure 2D). Analysis of curated gene sets (Supplemental Table 5) revealed that type I IFN response genes (*n* = 43) were upregulated after the first mRNA vaccine, whereas type II IFN response genes (*n* = 53) were not (Figure 2E and Supplemental Figure 4F). In contrast, both type I and type II IFN response genes were activated after the second mRNA vaccine and after Ad26.COV2.S vaccination. Notably, Ad26.COV2.S induced a significantly higher type I IFN response than the mRNA booster, whereas the type II IFN responses were comparable.

Together, these results show that mRNA vaccination elicits a modest type I IFN response after the first dose in myeloid cells, followed by a robust, broader IFN program after the second dose. The transcriptional IFN response to Ad26.COV2.S resembled that of the mRNA booster but was markedly stronger in magnitude, driven by the stronger type I IFN responses.

### The first mRNA dose induces a distinct type I IFN response state in myeloid cells.

**Figure 3:**
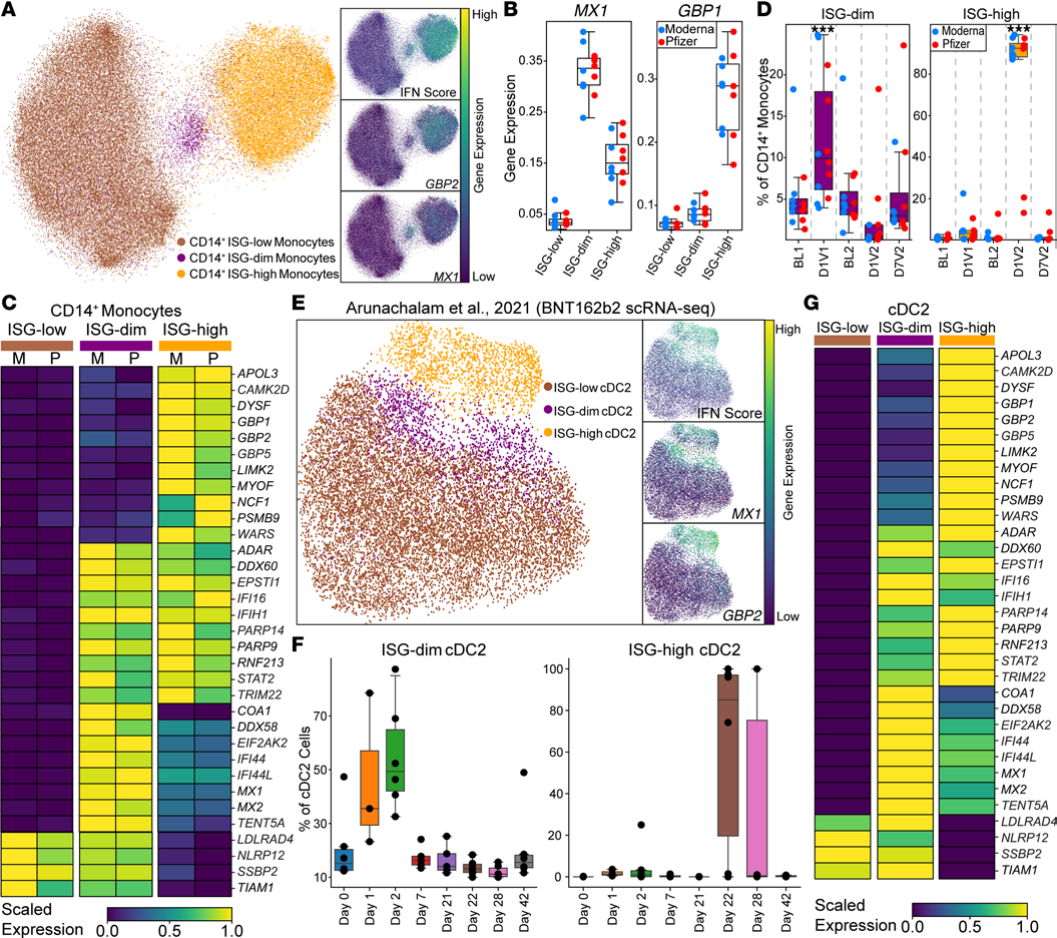
Distinct ISG states associated with primary and booster mRNA vaccination in CD14^+^ monocytes and DCs. (**A**) UMAPs highlight ISG states (ISG-low, ISG-dim, ISG-high) in CD14^+^ monocytes and demonstrate expression of marker gene. (**B**) Box plots show expression of *MX1* and *GBP1*. (**C**) Heatmap showing the expression of genes differentially expressed between 3 ISG states in Moderna (M) and Pfizer (P) vaccine recipients. (**D**) The proportion of ISG-dim and ISG-high cells within CD14^+^ monocytes across mRNA vaccines and time points. Note that the ISG-dim population expands specifically after the first mRNA vaccination. (**E**) UMAP highlights ISG states (ISG-low, ISG-dim, ISG-high) in cDC2s upon reanalysis of publicly available data. (**F**) The proportion of ISG-dim and ISG-high cells within cDC2s across time points. (**G**) The heatmap displays the expression levels of marker genes for ISG states in cDC2s. For **D**, statistical comparisons between time points were performed using the 1-sided Wilcoxon test: ***P < 0.001.

We next focused on myeloid cell responses to the first mRNA vaccination by performing a detailed analysis of CD14^+^ monocytes. This analysis revealed 3 distinct IFN-associated transcriptional states: ISG-low, ISG-dim, and ISG-high (Figure 3A and Supplemental Figure 5A). ISG-dim cells selectively expressed a subset of canonical type I IFN response genes, including antiviral effectors *MX1* and *MX2*; ISGs *IFI44*, *IFI44L*, and *ISG15*; and the pattern recognition receptor (PRR) *DDX58/RIG-I* (Figure 3, B and C, and Supplemental Table 6). In contrast, ISG-high cells displayed a broader IFN signature, including the upregulation of IFN-γ–induced guanylate-binding proteins (GBPs), the tRNA synthetase *WARS*, and immunoproteasome components such as *PSMB9* (*LMP2*) (22–24) (Figure 3C). Notably, the ISG-dim state expanded after the first mRNA dose; approximately 12% of CD14^+^ monocytes were in this state after BNT162b2 or mRNA-1273 vaccination, compared with approximately 5% at baseline (~2.36-fold increase) (Figure 3D).

We identified cell surface markers enriched in the ISG-dim and ISG-high states: *CD169* (*SIGLEC1*) and *CD64* (*FCGR1A*), respectively (Supplemental Figure 5B). Next, we performed flow cytometry on CD14^+^ monocytes from 6 mRNA-vaccinated donors to validate these transcriptional subsets at the protein level. Frequency of CD14^+^CD169^+^ cells, reflecting type I IFN responses, increased significantly after the first vaccination (*P* = 0.036) and reached its highest levels after the second dose (*P* = 0.014). In contrast, CD14^+^CD64^+^ cells, reflecting type II IFN responses, increased significantly only after the booster vaccination (*P* = 0.0007) (Supplemental Figure 5C), consistent with the transcriptomic patterns observed at corresponding time points (Supplemental Figure 5D). Notably, the ISG-dim transcriptional signature remained restricted to myeloid populations.

We reanalyzed cellular indexing of transcriptomes and epitopes by sequencing (CITE-Seq) data from DC-enriched PBMCs of 6 BNT162b2-vaccinated individuals (16) and confirmed the presence of all 3 ISG states (ISG-low, ISG-dim, ISG-high) in CD14^+^ monocytes in this independent cohort (Supplemental Table 3). Furthermore, we detected the same subsets in CD16^+^ monocytes and cDC2s (Figure 3E and Supplemental Figure 6A). In all 3 cell types, the ISG-dim state expanded on day 1 after the first vaccine, peaked at day 2, and declined by day 7 (Figure 3F and Supplemental Figure 6B). In contrast, the ISG-high state emerged on day 1 after the second vaccine (Figure 3F and Supplemental Figure 6B). Marker gene expression in this cohort mirrored our findings: ISG-dim cells highly expressed *MX1*, *MX2*, and *DDX58*, whereas ISG-high cells highly expressed a broader panel of ISGs, including IFN-γ–induced genes (Figure 3G and Supplemental Figure 6C).

Taken together, priming induced an early program (ISG-dim state), characterized by type I IFN response genes, while boosting elicited a robust response (ISG-high state), marked by broader activation of both type I and type II IFN response genes.

### Priming induces epigenetic activation of ISGF3 complex in CD14^+^ monocytes.

**Figure 4:**
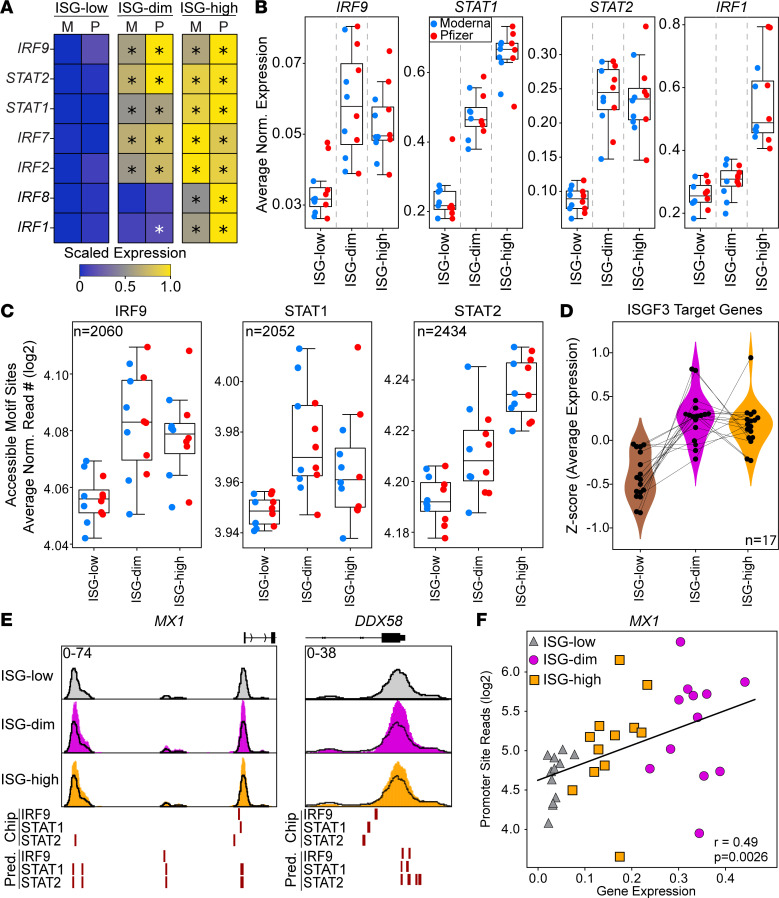
ISGF3 transcription factor complex activity increases in ISG-dim subset. (**A**) Heatmap displaying expression of key IFN response regulatory transcription factors across ISG subsets. (**B**) Average expression of the members of the ISGF3 complex (*IRF9*, *STAT1*, and *STAT2*) and *IRF1*. (**C**) Average chromatin accessibility at ISGF3 member binding sites across 3 ISG subsets using validated binding sites from a published study. (**D**) We calculated *z* scores of ISGF3 target genes (*n* = 17) based on the average expression of these genes in individual donors across ISG-defined states. (**E**) Genome browser views of *MX1* and *DDX58* loci across ISG states, highlighting predicted and validated (ChIP-Seq) binding sites for *IRF9*, *STAT1*, and *STAT2*. (**F**) Pearson’s correlation between promoter accessibility and gene expression for *MX1*.

Transcription factors (TFs) such as IFN regulatory factors (IRFs) and STATs are key regulators of IFN responses (25, 26). To determine whether the ISG-dim and ISG-high states exhibit distinct TF activity, we first examined the ISGF3 complex — the primary effector of type I IFN signaling (27, 28). The ISGF3 components STAT1, STAT2, and IRF9 were upregulated in both ISG-dim and ISG-high CD14^+^ monocytes (Figure 4, A and B). In contrast, IRF1 and IRF8, critical to type II IFN–driven programs (26, 29), were only induced in the ISG-high state (Figure 4, A and B).

To explore the epigenetic characteristics of these ISG states (Figure 3A), we analyzed chromatin accessibility in CD14^+^ monocytes. Using previously published ChIP-Seq data from human monocytes for STAT1, STAT2, and IRF9 (27), we identified validated ISGF3 binding sites (27) and assessed their accessibility in our ATAC-Seq data. ISGF3 binding sites were significantly more accessible in both ISG-dim and ISG-high monocytes compared with ISG-low monocytes (Figure 4C and Supplemental Figure 7A). Only 17 genes had binding sites at their promoters for all 3 ISGF3 TFs (Figure 4D and Supplemental 7B). These genes included *MX1*, *OAS1*, *OAS3*, and *DDX58* (RIG-I), markers of the ISG-dim state. The promoters of these genes exhibited increased chromatin accessibility in ISG-dim cells and harbored ISGF3 binding motifs (Figure 4, E and F, and Supplemental Figure 7B), further supporting the notion that they are direct ISGF3 targets.

ChromVAR analysis of IRF and STAT motifs, together with longitudinal transcriptomic data, demonstrated that the increased binding accessibility and expression observed for these TFs after the first mRNA vaccine dose was transient (Supplemental Figure 8A); no significant changes were detected at the second baseline prior to booster vaccination. Similarly, the changes observed upon booster vaccination (i.e., the ISG-high state) had resolved by day 7 after vaccination.

These analyses indicate that the transcriptional and epigenetic activation of the ISGF3 complex is a defining feature of the ISG-dim state induced by the priming by mRNA vaccines.

### Exposing monocytes to type I and type II IFNs recapitulates ISG-dim and ISG-high states, respectively.

**Figure 5:**
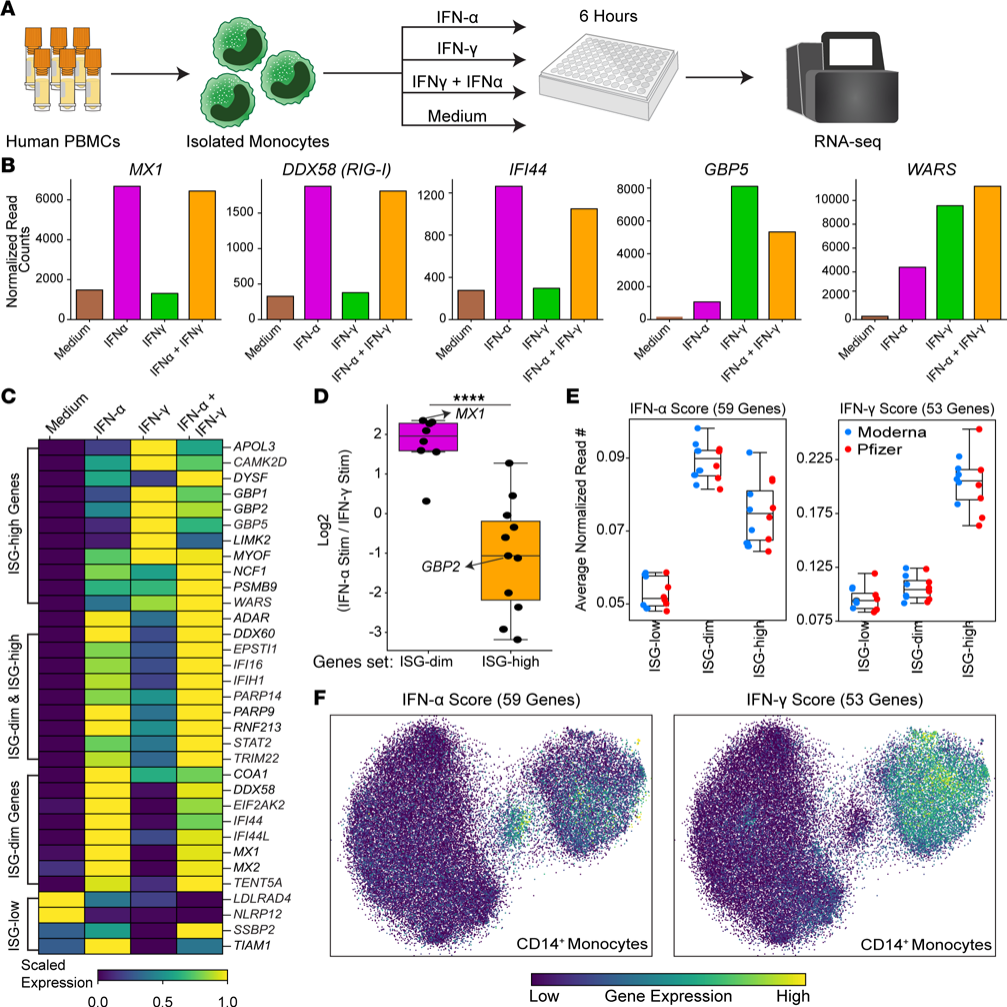
Monocyte stimulation with IFN-α and IFN-γ reproduce in vivo ISG states. (**A**) The experimental design involved generating bulk RNA-Seq data from isolated monocytes stimulated with either IFN-α or IFN-γ or their combination. (**B**) The expression of *MX1, DDX58, IFI44, GBP5,* and *WARS* upon stimulation. (**C**) The heatmap displays the expression levels of marker genes for ISG states in in vitro stimulated data. (**D**) The log_2_ fold change between IFN-α and IFN-γ stimulation experiments for markers of ISG-dim or ISG-high states. (**E**) Expression levels of genes in 3 ISG states for genes associated with IFN-α or IFN-γ stimulation response. Note that the ISG-dim state has a high IFN-α score, whereas ISG-high has high IFN-α and IFN-γ scores. (**F**) The UMAP shows the visualization of cells with high IFN-α or high IFN-γ scores. Note the colocalization of high IFN-α cells with the ISG-dim state and colocalization of high IFN-γ score cells with the ISG-high state. For **D**, statistical comparisons were performed using the Mann-Whitney *U* test: ****P < 0.0001.

We hypothesized that type I and type II IFNs drive the ISG-dim and ISG-high transcriptional states, respectively. Supporting this, the frequency of ISG-high CD14^+^ monocytes correlated strongly with elevated plasma IFN-γ levels after the second mRNA vaccine dose (*R* = 0.952, *P* = 5.38 × 10, Supplemental Figure 8B). To test whether IFN-α and IFN-γ are sufficient to induce the distinct ISG states observed in vivo, we stimulated human monocytes for 6 hours with IFN-α, IFN-γ, or both and performed RNA-Seq (Figure 5A). IFN-α stimulation selectively upregulated ISG-dim markers, including *MX1*, *DDX58*, and *IFI44*, whereas IFN-γ stimulation upregulated ISG-high markers including *GBP5* and *WARS* (Figure 5B).

Gene expression patterns after IFN-α or IFN-γ treatment closely mirrored the in vivo ISG-dim and ISG-high transcriptional profiles, respectively. ISG-dim–specific genes were robustly induced by IFN-α, while ISG-high–specific genes were preferentially activated by IFN-γ (Figure 5, C and D). To further relate these in vitro gene programs to in vivo single-cell clusters, we derived IFN-α and IFN-γ gene signatures from the stimulation experiments (Figure 5E and Supplemental Table 7). UMAP projection of these gene sets revealed distinct localization: ISG-dim monocytes predominantly expressed the IFN-α response gene set, while ISG-high monocytes expressed both IFN-α and IFN-γ response gene sets (Figure 5F).

Together, these data confirm that type I IFN signaling is sufficient to drive the early priming response (ISG-dim state) induced by the first mRNA vaccine dose, whereas type II IFN is required to establish the broader boosting response (ISG-high state) observed after the second dose.

## Discussion

In this study, we identified 2 distinct IFN programs in human myeloid cells (ISG-dim and ISG-high) that are differentially engaged by mRNA priming, mRNA boosting, and adenovirus vaccination. To our knowledge, this is the first head-to-head, single-cell multiomic comparison of all 3 FDA-approved COVID-19 vaccines in SARS-CoV-2–naive individuals. This dataset, collected in the unprecedented context of a global pandemic, captures the earliest programming events in human myeloid cells in response to a novel antigen delivered through mRNA and adenovirus vaccine platforms.

Our data reveal that mRNA priming rapidly (1–2 days after vaccination) induces a transient ISG-dim state in approximately 10% of myeloid cells, driven by type I IFN signaling, whereas boosting and single-dose adenovirus vaccination elicit a more robust ISG-high program involving both type I and type II IFNs. Although we did not observe correlations between early transcriptional changes or IFN-γ levels and IgG titers after the first vaccination, IFN-γ protein levels after the booster correlated significantly with IgG titer increases, consistent with previous reports. These findings suggest that IFN responses during the booster may influence the magnitude of the humoral response. The ISG-high response observed after booster vaccines aligns with prior reports showing that mRNA boosters induce markedly higher IFN-γ cytokine levels and increased expression of ISGs, likely stemming from memory CD4^+^ and CD8^+^ T cells primed by the first dose (9, 12, 17, 30–32). In addition, murine studies demonstrate that impaired type I IFN production, via MDA5 or IFNAR deficiency or IFN-β blockade, significantly diminishes antigen-specific CD8^+^ T cell responses, underscoring the importance of early innate signaling in vaccine-induced immunity (9, 32). Although prior reports described strong IFN responses after the booster dose, there were limited changes reported after the first mRNA dose (13, 17, 18, 30), likely because these studies have typically focused on bulk transcriptional changes. Our work extends these observations by providing cell type–specific resolution, mechanistic separation of IFN-driven states, and direct comparison across vaccine platforms.

The transcriptional and epigenetic differences between these 2 states suggests that the innate immune system is differently reprogrammed between priming and boosting. The ISG-dim state was marked by early chromatin accessibility and transcriptional activation of ISGF3 complex components (STAT1, STAT2, IRF9), which in turn regulated canonical type I IFN response genes and ISG-dim markers *MX1* and *DDX58*. Additional IRF1 and IRF8, typically associated with type II IFN signaling (25, 33), were activated in the ISG-high state. Although we observed strong early epigenetic remodeling, these changes did not persist: the ISG-dim epigenetic state was not detected at the second pre-boost baseline, in line with prior reports showing limited innate immune memory following mRNA vaccination (17, 18, 34). Nevertheless, we cannot rule out the possibility of longer-term reprogramming in bone marrow progenitors (35–37) or the migration of epigenetically modified myeloid cells to peripheral tissues, either in their existing state or after differentiation into antigen-presenting cells such as DCs or macrophages. Moreover, sustained epigenetic changes may require repeated exposures or may be easier to observe in in vitro cultured monocyte-derived macrophages than in freshly isolated blood cells (38).

In vitro stimulation of myeloid cells with IFN-α alone or in combination with IFN-γ reproduced the ISG-dim and ISG-high transcriptional programs induced in vivo, respectively. Type I IFNs driving the ISG-dim state might be produced either via autocrine signaling by myeloid cells sensing mRNA vaccine components through pattern recognition receptors, such as MDA5 (9), or through paracrine signaling from other cell types, such as fibroblasts (32) or other immune cell types at the site of injection (32, 39, 40). The IFN-γ required to elicit the ISG-high state after the booster vaccine is most likely produced by antigen-specific T cells, which expand after booster vaccination (9, 12, 15, 18, 31). Yet, contributions from innate lymphoid cells, including NK cells, cannot be ruled out (41–43). The precise vaccine component(s) triggering these responses remain unclear. Although the mRNA backbone includes nucleoside modifications (e.g., m1Ψ) designed to evade immune recognition, dsRNA contaminants or the lipid nanoparticle formulation may retain immunostimulatory capacity (9, 32, 44, 45). Future work is needed to dissect which vaccine components drive the early IFN production in vivo.

In line with previous studies, mRNA vaccines elicited stronger humoral responses than the single-dose Ad26.COV2.S vaccine, with antibody titers significantly increasing after the second mRNA dose (46–48). Surprisingly, the Ad26.COV2.S vaccine triggered the most robust IFN response among all platforms, indicating that the magnitude of ISG programs does not necessarily correlate with humoral immunity. It is possible that the memory response to the adenoviral vector diverts the immune response away from SARS-CoV-2–specific targets (49, 50). The 2 mRNA vaccines used in our study, BNT162b2 and mRNA-1273, differ substantially in antigen dose (100 μg vs. 30 μg, respectively), yet we observed no major differences in their transcriptional, epigenetic, or antibody responses. Previous studies conducted in larger cohorts have reported significantly higher antibody titers and greater efficacy for the mRNA-1273 vaccine compared with the BNT162b2 vaccine (51–54). The absence of such differences in our study might stem from the relatively small sample size (*n* = 6 per group), which limits our ability to detect platform-specific effects. Larger cohorts will be needed to determine whether the antibody differences observed between these vaccines are accompanied by corresponding transcriptomic/epigenomic differences, and whether such differences arise from the mRNA dose or differences in vaccine formulation, such as the lipid nanoparticle composition used in each vaccine (39, 55, 56).

The use of multiple doses is a widely adopted strategy to achieve optimal immunity. However, the systemic dynamics of immune responses elicited by prime–boost vaccination remain incompletely understood for most 2-dose vaccines due to the lack of longitudinal genomic studies. Our findings show that the first and second doses of mRNA COVID-19 vaccines induce distinct innate immune responses, with substantially higher IgG responses observed after the booster dose. Similar patterns have been reported for other 2-dose vaccines, including MVA monkeypox, Ebola, M72/AS01 tuberculosis, and RZV shingles vaccines, where booster doses elicit higher antibody titers and enhanced cellular immunity (57–64). A diverse set of cytokines is induced as soon as 1 day after the primary dose for several of these vaccines (61, 65–67), including IP-10 (CXCL10), IL-6, CCL3, and CCL4 (61, 65–68). Although most of these studies did not generate post-booster cytokine or gene expression data, stronger IFN-γ induction after booster vaccination was observed for MVA vaccines in cynomolgus macaques and CMV mRNA vaccines in humans, consistent with our observations. Additionally, convalescent and/or seroprotected individuals secrete IFN-γ at higher levels after the primary dose compared with non-convalescent individuals (30). Notably, single-cell transcriptomic data for these vaccines are currently lacking, which limits direct comparison of the underlying innate programs induced by these 2-dose vaccines and the COVID-19 mRNA vaccines studied here.

Our findings indicate that the IFN responses observed in myeloid cells depend both on the vaccine platform and on whether individuals received a priming or booster mRNA dose. The positive correlation between post-booster IFN-γ levels and IgG titers suggests that stronger IFN activation may contribute to more robust humoral immunity, although whether such differences translate into improved protection or enhanced durability remains unclear. Since our cohort was relatively small, we were unable to link transcriptional or epigenetic signatures with epidemiological variables such as age or baseline immune status, both of which likely modulate innate and adaptive vaccine responses. Future studies are needed to determine whether the ISG states we observed are altered in older adults or immunocompromised individuals who often exhibit suboptimal immunity. Studies in mice and organoid models have reported that using IFN-α as an adjuvant can improve vaccine responses (69, 70). Moreover, IFN-α–treated immunocompromised individuals show enhanced antibody responses (71). Together, these data suggest that the magnitude of early IFN responses might contribute to the strength and durability of humoral immunity (9, 32) and might inform the design of next-generation vaccines.

This study provides the first detailed characterization to our knowledge of myeloid cell responses to priming and boosting with mRNA COVID-19 vaccines, leveraging the unique conditions created by the pandemic, a novel antigen delivered through a novel platform. Our findings underscore the pivotal role of early type I IFN signaling in shaping downstream immune responses. How these early myeloid programs influence adaptive immunity, particularly in older or immunocompromised individuals, remains an open and clinically relevant question. As mRNA technology continues to expand into vaccines for influenza, RSV, CMV, and other emerging threats, understanding how priming and boosting differentially program innate immunity will be essential for maximizing long-term protection. The framework we establish here can inform the design and clinical evaluation of these new vaccines, ensuring they elicit the most protective IFN-driven responses in diverse populations.

### Limitations

This study offers a single-cell resolution view of mRNA vaccine–induced IFN responses, but several limitations should be acknowledged. First, our cohort size was relatively small, and the Ad26.COV2.S group included only 3 donors, which limited our ability to detect subtle platform-specific differences. Second, although total cell numbers were sufficient for robust major cell-type annotation, the relatively low recovery of rare myeloid subsets, particularly DC subsets, limited our ability to perform detailed subset-level analyses. Therefore, we reanalyzed an independent dataset enriched for DCs to validate and confirm our findings. Third, our focus was restricted to circulating immune cells; missing tissue-resident or lymphoid compartment responses that contribute to early IFN signaling. Finally, although in vitro stimulation experiments supported the role of type I and type II IFNs in shaping ISG states, they did not fully recapitulate the complexity of in vivo cytokine and cellular interactions during vaccination.

## Methods

### Sex as a biological variable.

The study cohort included both male and female participants, with a majority being female. Sex was not considered as a biological variable in the study.

This study was conducted following approval by the UConn Health Center IRB (21-149J-1). This prospective, single-site, multicohort observational study was designed to determine how vaccine formulations and participant age affect immunologic responses to the SARS-CoV-2 vaccine and to uncover the molecular signatures elicited by novel COVID-19 vaccines. A total of 31 healthy adults aged 21 years and older (21–74 years old; mean age 43.7 ± 13.3; 71% female, 29% male; CMV status) who had received an influenza vaccination for the 2020–2021 season but had not yet received a SARS-CoV-2 vaccine were enrolled.

Blood samples were collected from the participants at 8 study visits over a 1-year period for mRNA vaccines: baseline (day –4 to day 0 prevaccination), 1 day after first vaccination, day 7 after first vaccination, baseline 2 (days 25–28; prior to vaccination dose 2), 1 day after dose 2, 7 days after dose 2, D42V2 (day 70 ± 3 days), and D154V2 (day 180 ± 10 days). For Ad26.COV2.S. vaccination, blood samples were collected at 6 study visits over a 1-year period: baseline (day –4 to day 0 prevaccination), 1 day after first vaccination, day 7 after first vaccination, day 35 (days 32–35 after vaccination), day 70 (day 70 ± 3 days), and day 180 (day 180 ± 10 days). Additionally, blood samples were collected at approximately 365 days after vaccination for participants vaccinated with mRNA or Ad26.COV2.S; however, these samples were not used in the subsequent experiment and analysis.

For all participants, medical history, demographics, vitals (blood pressure, heart rate, temperature), height, weight, BMI, and medication use were collected in the REDCap platform. For individuals 65 years and older, frailty assessments were also performed. For each participant, we reported age, sex, ethnicity, and CMV status (Supplemental Table 1).

### PBMC isolation.

Plasma was separated from the blood collected in ACD tubes by centrifugation, whereas PBMCs were isolated by Lymphoprep (STEMCELL Technologies) gradient centrifugation within 1 hour after collection of samples.

### ELISA.

For SARS-CoV-2 spike–specific ELISA assays, Nunc MaxiSorp flat-bottom 96-well plates (Invitrogen) were coated with 2 μg/mL of recombinant trimeric full-length spike protein (50 μL per well, produced in HEK293T cells using pCAGGS plasmid vector containing Wuhan-Hu-1 spike glycoprotein gene obtained through BEI Resources, NR-52394) in bicarbonate buffer overnight at 4°C. Plates were washed 3 times with 1× PBS plus 0.1% v/v Tween-20 (PBST). Subsequently, plates were blocked with 100 μL per well of blocking solution (5% nonfat dry milk in PBST) for 1 hour at room temperature (RT). After removal of blocking buffer, hamster sera were serially diluted in blocking solution starting at 1:100 dilution and incubated for 1.5 hours at RT. After washing plates with PBST, 50 μL of HRP-conjugated goat anti-human IgG cross-adsorbed antibody (Southern Biotech, 2040-05) was added at 1:5,000 dilution. Plates were incubated for 1 hour at RT and washed 3 times with PBST. Finally, 100 μL tetramethyl benzidine (TMB; optEIA, BD Biosciences, 555214) substrate was added and incubated at RT until blue color was developed. The reaction was stopped with 50 μL 1M H_2_SO_4_, and absorbance was recorded at 450 nm and 650 nm as a reference. An average of OD_450_ values for blank wells plus 3 standard deviations was used to set a cutoff value for each plate.

### Determination of CMV seropositivity.

CMV serostatus was determined using CMV IgG ELISA kits (Aviva Systems Biology). Plasma samples were thawed at RT, diluted, and analyzed according to the manufacturer’s recommendations. Calculation of the results was done based on the appropriate controls included in the kit.

### Pilot sequencing.

A pilot sequencing analysis was conducted to determine which visits to focus on and sequence more of. One donor for each vaccine was randomly chosen, and 8 visits (baseline 1, days 1 and 7 after dose 1, baseline 2, days 1 and 7 after dose 2, and D42V2, D154V2) of mRNA recipients and 6 visits (baseline and days 1, 7, 35, 70, and 180) of the Johnson & Johnson recipients were sequenced and analyzed. D42V2 refers to 42 days after the second vaccination (V2), corresponding to 70 days after the first vaccination (V1), while D154V2 refers to 154 days after the second vaccination (V2), corresponding to 180 days after the first vaccination (V1). Based on this preliminary analysis, 5 visits (baseline 1; day 1, vaccine 1; baseline 2; day 1, vaccine 2; day 7, vaccine 2) of mRNA vaccine recipients and 3 (baseline and days 1 and 7) of Johnson & Johnson recipients were chosen for sequencing. 

### ELLA microfluidic immunoassay.

The level of 4 cytokines (IFN-γ, IL-8, IL-10, and TNF-α) and 2 chemokines (CCL2 and CXCL10) in longitudinal plasma samples was quantified using Simple Plex assays run on ELLA microfluidic immunoassay platform (ProteinSimple). Plasma samples, diluted 1:1 in sample diluent, were added on the ELLA microfluidic cartridges (human SPCKE-06 cartridge for IL-8/CXCL8, CCL2/JE/MCP-1, IL-10, CXCL10/IP-10/CRG-2, IFN-γ 3rd generation, TNF-α 2nd generation) according to the manufacturer’s instructions. The ELLA system reported the mean values of the built-in triplicate analysis for each analyte using the manufacturer-calibrated standard curve within 90 minutes.

### DOGMA-Seq library preparation and sequencing.

Cryopreserved PBMCs were thawed, washed, and stained with barcoded hashing and phenotyping TotalSeqA antibodies (BioLegend) as previously described for DOGMA-Seq (19). Stained cells were fixed in 0.1% formaldehyde for 5 minutes at RT and quenched with glycine solution to a final concentration of 0.125 M before washing twice in PBS via centrifugation at 400*g*, 5 minutes, 4°C. Cells were subsequently treated with lysis buffer (10 mM Tris-HCl pH 7.4, 10 mM NaCl, 3 mM MgCl_2_, 0.1% NP40, 1% BSA) for 3 minutes on ice, followed by adding 1 mL of chilled wash buffer (10 mM Tris-HCl pH 7.4, 10 mM NaCl, 3 mM MgCl_2_, 1% BSA). Cells were harvested and diluted in 1× diluted nuclei buffer (10x Genomics) and filtered through a 40 μm Flowmi cell strainer before counting using trypan blue and a Countess II FL Automated Cell Counter. From there on, the 10x Multiome protocol was followed with the following modifications: (a) during preamplification PCR, 1 μL of 0.2 μM additive primer (antibody derived tags [ADT] and hashtag oligo [HTO]) was added. ADT add = 5-CCTTGGCACCCGAGAATT*C*C-3 HTO add = 5-GTGACTGGAGTTCAGACGTGTGC*T*C-3; and (b) 35% of the preamplified sample was used to amplify and index protein tags.

GEX, ATAC, ADT, and HTO libraries were pooled and sequenced on an Illumina NovaSeq S4 flow cell.

### FASTQ file processing and read alignment.

Reads for scATAC-Seq and scRNA-Seq libraries were mapped and aligned to the reference human genome (GRCh38) using Cell Ranger Arc software (2.0.1). The individual Cell Ranger outputs were aggregated without normalization using Cell Ranger. ADT and HTO reads were processed using kallisto and bustools pipeline (72, 73). Sample demultiplexing was done using HTODemux function from Seurat (74).

### DOGMA-Seq analysis.

scRNA-Seq, scATAC-Seq, and ADT data were processed using Muon and Scanpy libraries (75, 76). Clustering and cell-type annotations were done using scRNA and ADT data. scRNA-Seq data was total normalized. ADT data was centered log-ratio normalized and then total normalized. Highly variable genes were calculated from the scRNA data, and all ADTs except the controls were considered highly variable. After independent normalization, scRNA and ADT data were concatenated, and the rest of the pipeline was performed using highly variable features. After cell-type annotations were finalized, only scRNA data were used to discover the ISG^+^ subset. Batch correction was performed with BBKNN (77). While doing PBMC-level annotations, where ADTs are involved in clustering, the “pool” variable was used as the batch key, and for fine-level annotations, “library” was used.

scATAC-Seq data were processed, and quality control metrics were assessed using SnapATAC2 from 10x fragment files (78). Cell multiplets were identified using AMULET with an FDR threshold of less than 5% and excluded from downstream analyses (79). Cells with fewer than 1,000 fragments or a transcription start site enrichment score below 10 were removed. The intersection of the cells in scATAC data and scRNA data was taken, and the labels of cell subsets were transferred to scATAC data. Peak calling was performed on BAM files for each cell subset using MACS3 with the parameters --call-summits -q 0.05 --nomodel --extsize 200 --shift -100 (80). The peak matrix for each cell was constructed using SnapATAC2 with a paired-insertion counting strategy (81).

### Differential gene expression analysis.

Three distinct CD14^+^ monocyte subsets were identified, ISG-low, ISG-dim, and ISG-high, as follows: Mean expression of each gene per donor/subset was calculated, and distributions were compared using a 2-tailed paired *t* test with SciPy library. *P* values were adjusted using the statsmodels library and the Benjamini-Hochberg method. Genes that were expressed in more than 10% of cells within any cell subset of interest and expressed in less than 90% of all cells in the analysis were considered in this analysis. The DESeq2 was used to conduct DEG analysis, comparing each visit to the corresponding baseline (82). Prior to analysis, genes expressed in fewer than 1% of cells across all cell subsets of interest were excluded.

### TF motif binding site accessibility.

Iterative overlap peaks were identified using the same MACS3 output for each cell subset, and a peak matrix was generated using SnapATAC2 by extending each peak center by ±250 bp. The TOBIAS (83) (v0.14.0) pipeline was utilized to predict motif binding sites from HOCOMOCO (84). To identify the experimental binding sites, publicly available IRF9, STAT1, and STAT2 ChIP-Seq data from the THP-1 cell line were utilized (27). The FASTQ files were processed and analyzed using the MACS3 pipeline (80). The “bdgpeakcall” function from the MACS3 function was used to call peaks for each TF with a threshold set to yield approximately 100,000 peaks for downstream analysis. Regions present in fewer than 1% of cells across all CD14^+^ ISG subsets that overlapped with ChIP-Seq–derived binding sites were selected. ISGF3 binding sites were identified by intersecting IRF9, STAT1, and STAT2 binding sites within scATAC-Seq–accessible regions. ChromVAR was employed to assess motif binding site accessibility deviations for each cell subset using HOCOMOCO motifs (85).

### Cell proportion analysis.

Cell proportions of a subset within a superset were done by simply calculating the percentage of the subset of interest within the superset (all PBMCs, the cell type, etc.). Statistical comparisons were done using a 1-tailed Wilcoxon test.

### Reanalysis of published data.

Publicly available DC-enriched CITE-Seq data (16) was reanalyzed using Scanpy. ISG-low, ISG-dim, and ISG-high subsets were identified in monocytes and DCs.

### In vitro monocyte stimulation.

Human monocytes were purified from PBMCs isolated from healthy donors using Dynabeads untouched human monocyte kit (Thermo Fisher Scientific). The purity of CD14^+^ cells by flow cytometry analysis was greater than 98%. Purified monocytes, resuspended in complete RPMI medium (RPMI medium 1640 supplemented with l-glutamine, sodium pyruvate, HEPES buffer, penicillin-streptomycin, and 10% v/v FBS), were seeded at a density of 1 × 10^6^/mL/well in 96-deep well plates (Thermo Fisher Scientific) and stimulated for 6 hours as follows: 100 pg/mL recombinant human IFN-γ (R&D Systems), in the absence or presence of 400 ng/mL of anti-IFN-γ-IgG neutralizing antibody (InvivoGen, clone H7WM120), 100 U/mL recombinant human IFN-α (PBL Assay Science), in the absence or presence of 400 ng/mL of anti-IFN-α-IgG neutralizing antibody (Invivogen, clone H7WM116), 100 pg/mL IFN-γ plus 100 U/mL IFN, or medium control (unstimulated). At the end of incubation, monocytes were collected by centrifugation, and the cell pellets were resuspended in RLT buffer (QIAGEN) for total RNA extraction and processing for bulk RNA-Seq, as described below.

### Flow cytometry.

For analysis of the CD14^+^ compartment, PBMCs isolated from the blood collected at baseline, day 1 after the first vaccine, and day 1 after the second vaccine (day 1, vaccine dose 2) were stained with fluorochrome-labeled antibodies against the following surface markers: CD14 BV711 (clone M5E2, BD Biosciences, 1:100), CD64 BV421 (clone 10.1, BioLegend, 1:100), and CD169 PE-Cy7 (clone 7-239, BioLegend, 1:100). The stained cells were acquired using a BD Biosciences LSR Fortessa X-20 flow cytometer and analyzed with FlowJo V9.9.6 software.

### Bulk RNA-Seq of monocytes and analysis.

RNA-Seq libraries were prepared with an mRNA Library Prep kit (Watchmaker Genomics) according to the manufacturer’s instructions. Briefly, 100 ng total RNA was used as input and 2 μL of 1:1,000 dilution of External RNA Controls Consortium (Thermo Fisher Scientific) was spiked into each sample. First, poly A RNA was isolated from total RNA using oligo-dT magnetic beads. Purified RNA was then fragmented at 85°C for 10 minutes, targeting fragments in the range of 300–350 bp. Fragmented RNA was reverse-transcribed with an incubation of 25°C for 10 minutes, 42°C for 15 minutes, and an inactivation step at 70°C for 15 minutes. This was followed by second-strand synthesis and A-tailing at 42°C for 5 minutes and 62°C for 10 minutes. A-tailed, double-stranded cDNA fragments were ligated with xGen UDI-UMI adapters for Illumina (IDT). Adapter-ligated DNA was purified using KAPA Pure beads (Roche). This was followed by 12 cycles of PCR amplification and another clean up. Purified libraries were sequenced on NextSeq 2000 (Illumina), generating paired-end reads of 150 bp. Paired-end reads were preprocessed with adapter trimming and quality filtering using Cutadapt (86). Reads were aligned to the reference transcriptome (GENCODE v38) and quantified using Salmon (v1.10.3) with positional bias corrections and fragment GC bias (87). The gene level counts were generated using tximport (v1.34.0) (88). The counts were normalized with TMM using edgeR (v4.4.2) (89).

### ISG score calculations and density estimates.

IFN scores were calculated by taking the mean of genes from the IFN modules of BloodGen3Module (21). The ISG score density estimates were done using kernel density estimates via the Seaborn library’s kdeplot function. In scATAC-Seq analysis, genomic regions were prefiltered to retain those present in at least 1% of cells across all analyzed subsets. Smooth quantile normalization was performed using SNAIL (90) across each donor, followed by log_2_ transformation of read counts. The regions were annotated to their nearest gene using HOMER (91) and overlapped with genes from IFN modules from BloodGen3Module. The mean accessibility of the genomic regions was calculated to derive the epigenetic ISG score. Type I and type II IFN scores were calculated using the manually curated list of IFN genes (Supplemental Table 5). The IFN-α and IFN-γ gene lists were generated based on the fold change between medium and each other (Supplemental Table 7).

### Statistics.

Antibody titer level and cytokine-level comparisons between visits were done using a 2-tailed paired *t* test. Statistical comparisons of gene expression and chromatin accessibility from scRNA-Seq and scATAC-Seq were performed using a 2-tailed paired *t* test between visits at the donor level. *P* values less than 0.05 were considered significant.

### Study approval.

This study was conducted following approval by the UConn Health Center IRB (21-149J-1). Participants who were scheduled to receive an mRNA-based SARS-CoV-2 vaccine authorized for emergency use by the FDA as part of the federal SARS-CoV-2 vaccination program were enrolled. After a pre-telephone screening, participants provided written consent at baseline visit 1 or by e-consent prior to the visit.

### Data availability.

Raw read data from DOGMA-Seq and RNA-Seq experiments are in the NCBI’s database of Genotypes and Phenotypes (https://dbgap.ncbi.nlm.nih.gov/home/) under accession phs004038. The processed data can be viewed and explored at https://ucarlab.github.io/Covid19VaccineResponses/

Values for all data points in graphs are reported in the Supporting Data Values file.

### Code availability.

The scripts used for data processing and figure generation are available on GitHub (https://github.com/UcarLab/Covid19VaccineResponses; commit ID 73b8d1e51ccae2fe60a440d4162a61e257d4f6e9).

## Author contributions

Both co–first authors contributed equally, and the order of the co–first authorship was determined alphabetically by last name. JB, GAK, and DU designed the study and raised the funds. GAK and LKP coordinated the clinical sample collection. GE and YYY led the data analyses. MS, AGS, KH, and SJ generated and interpreted the antibody titer data. EPM generated DOGMA-Seq data. RM processed blood samples and generated cytokine data. YYY, GE, DU, and JB wrote the paper. All authors revised the manuscript and helped with data interpretation.

## Funding support

This work is the result of NIH funding, in whole or in part, and is subject to the NIH Public Access Policy. Through acceptance of this federal funding, the NIH has been given a right to make the work publicly available in PubMed Central.

- NIH grant R01 AI142086 (to DU, JB, and GK), U19 AI135972 (to AGS), U01 AI165452 (to DU, AGS, and GK), R01 AI160706 (to MS), and P30 AG067988 (to GK).

## Supplementary Material

Supplemental data

ICMJE disclosure forms

Supporting data values

## Version 1 02/23/2026

Electronic publication
