# Supplementary material for: Dose-dependent IFN programs in myeloid cells after mRNA and adenovirus COVID-19 vaccination
Source: JCI Insight. 2026 Feb 23;11(4):e199245. doi: 10.1172/jci.insight.199245 (PMC12956017; doi:10.1172/jci.insight.199245)
Supplement: Supplemental data [file jciinsight-11-199245-s179.pdf]

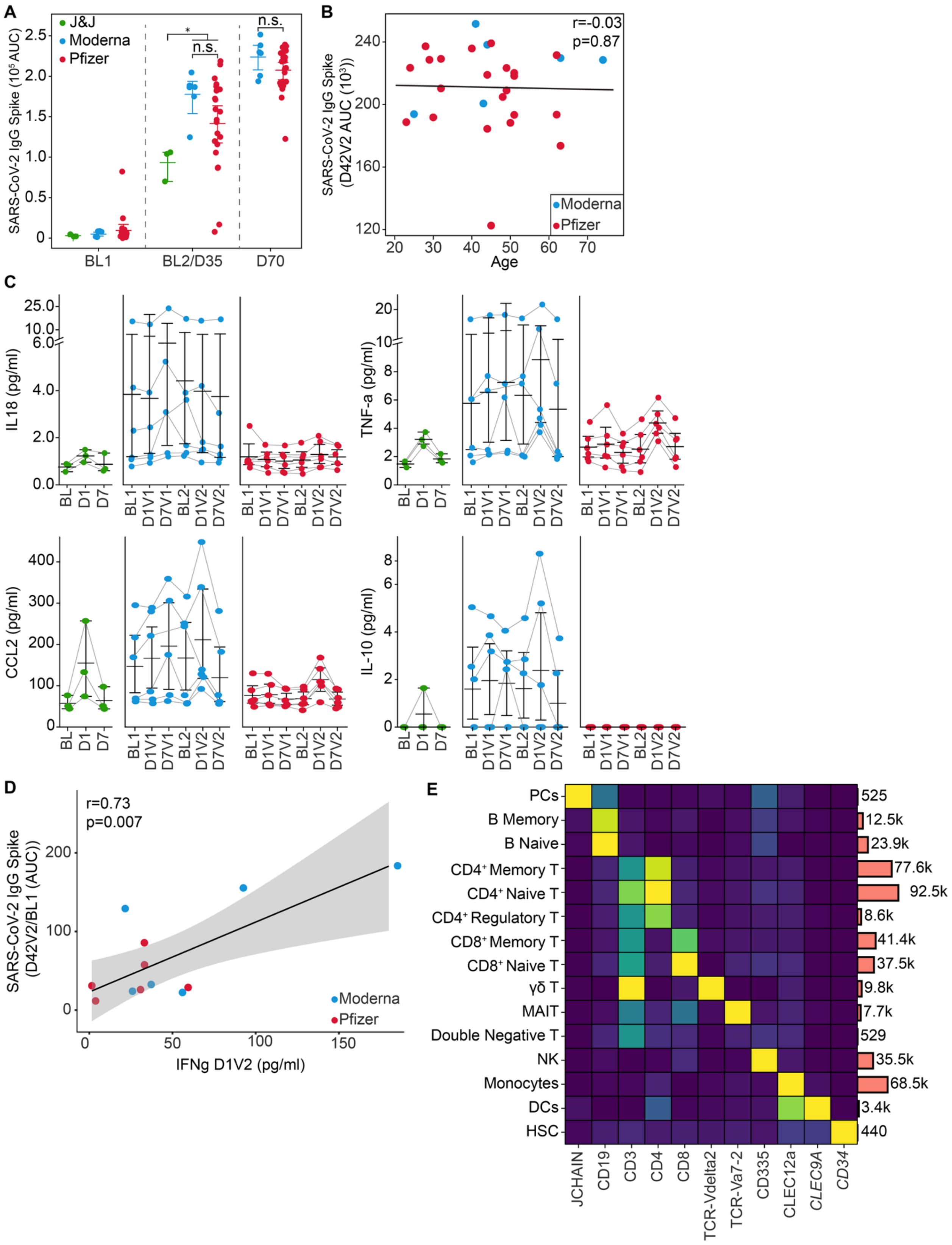

A
B
C
D
E
J&J
Moderna
Pfizer
SARS-CoV-2 IgG Spike (10⁵ AUC)
BL1
BL2/D35
D70
n.s.
*
SARS-CoV-2 IgG Spike (D42V2 AUC (10³))
r=-0.03
p=0.87
Age
IL18 (pg/ml)
TNF-a (pg/ml)
CCL2 (pg/ml)
IL-10 (pg/ml)
BL
D1
D7
BL1
D1V1
D7V1
BL2
D1V2
D7V2
SARS-CoV-2 IgG Spike (D42V2/BL1 (AUC))
r=0.73
p=0.007
IFNg D1V2 (pg/ml)
PCs
B Memory
B Naive
CD4+ Memory T
CD4+ Naive T
CD4+ Regulatory T
CD8+ Memory T
CD8+ Naive T
γδ T
MAIT
Double Negative T
NK
Monocytes
DCs
HSC
525
12.5k
23.9k
77.6k
92.5k
8.6k
41.4k
37.5k
9.8k
7.7k
529
35.5k
68.5k
3.4k
440
JCHAIN
CD19
CD3
CD4
CD8
TCR-Vdelta2
TCR-Va7-2
CD335
CLEC12a
CLEC9A
CD34

Supplementary Figure 1: Antibody titer and cytokine level after adenovirus and mRNA vaccination

(A) IgG titers against SARS-CoV-2 spike protein measured by ELISA. mRNA vaccines elicit higher antibody titer compared to J&J. (**B)** The correlation plot with age and antibody titer for mRNA vaccines. Pearson's correlation was used to calculate statistics. The Pearson correlation analysis examining the relationship between IFN-γ protein levels and IgG titers after booster vaccination in mRNA-vaccinated donors. **(C)** IL-8, TNFα, CCL2, and IL-10 cytokine levels, quantified by ELLA. **(D)** The Pearson correlation analysis examining the relationship between IFN-γ protein levels and IgG titers fold change (D42 vs BL1) in mRNA-vaccinated donors. **E)** The heatmap shows the marker genes for single-cell annotations. **(A)** The statistical analysis is conducted between all mRNA vaccine recipients (Moderna and Pfizer) and adenovirus vaccine recipients. Statistical comparisons were performed using the Mann-Whitney test: n.s.: non-significant, *$P < 0.05$, **$P < 0.01$, ***$P < 0.001$, ****$P < 0.0001$.

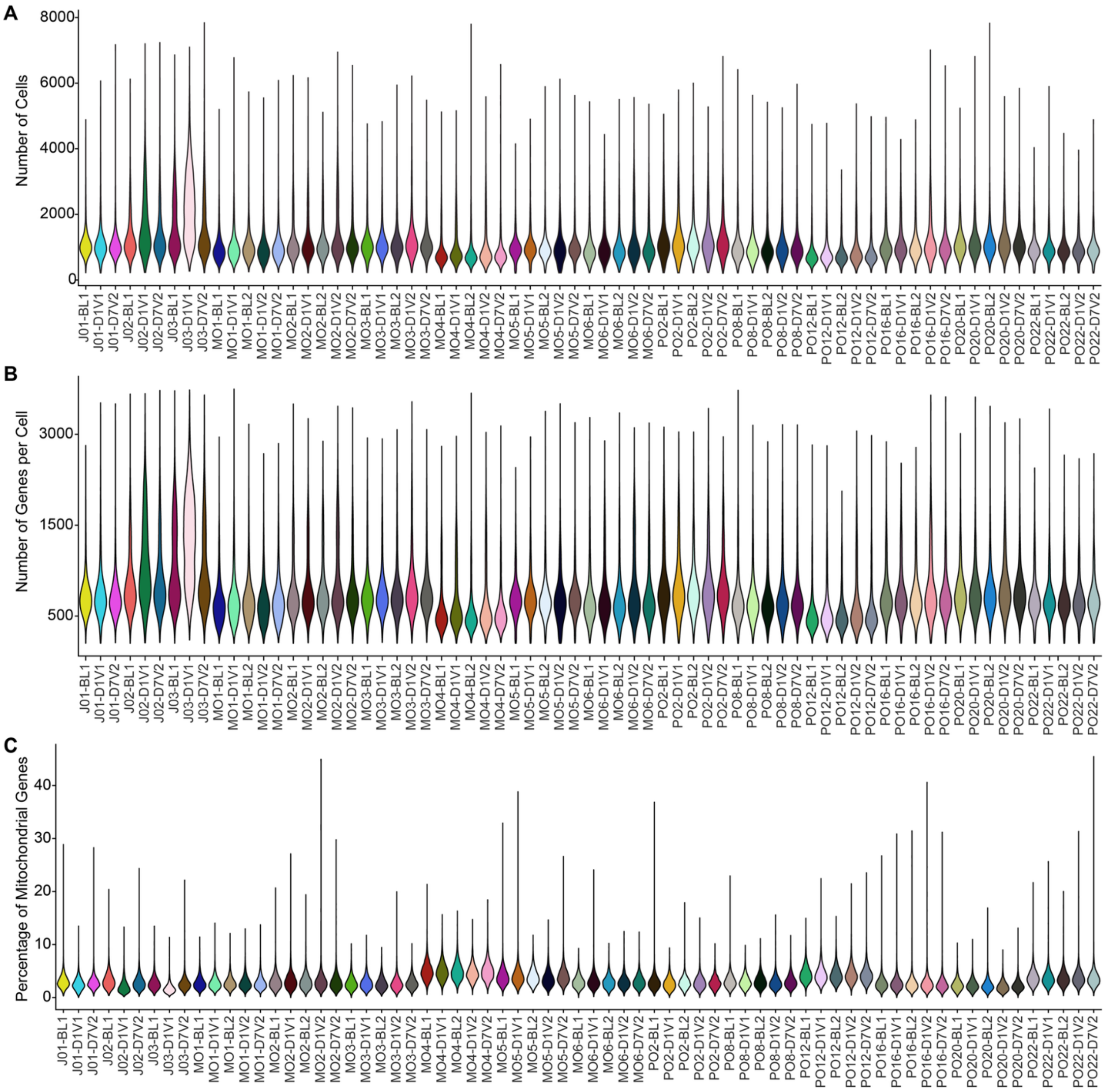

Supplementary Figure 2: The statistical and quality metrics used in RNA-seq pre-processing

(A) The number of cells per donor and time point is given as a violin plot. **(B)** The number of genes per cell for each donor and timepoint is given as a violin plot. **(C)** Mitochondrial gene percentage is given as a violin plot.

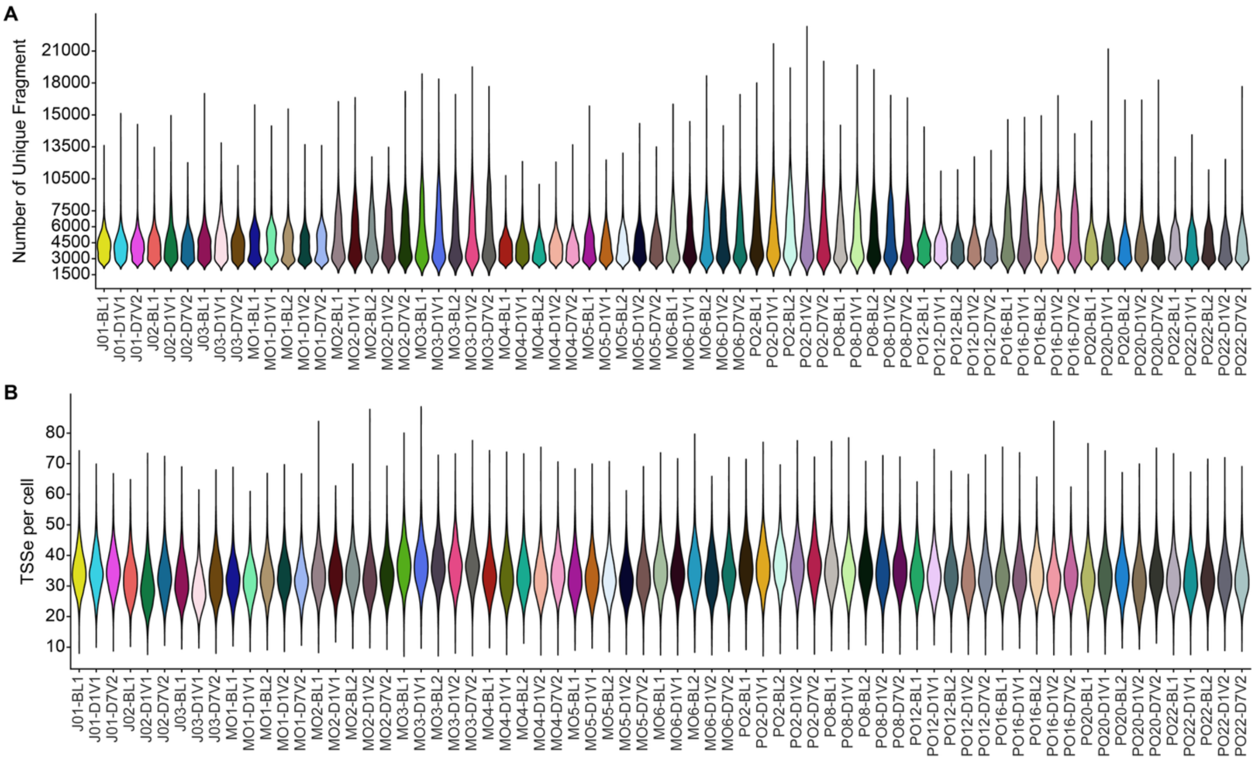

Supplementary Figure 3: The statistical and quality metrics used in ATAC-seq pre-processing, and the number of cells within the myeloid population.

(A) The number of unique fragments per donor and time point is given as a violin plot. **(B)** The Transcription Start Site enrichment score (TSSe) for each donor and time point is given as a violin plot.

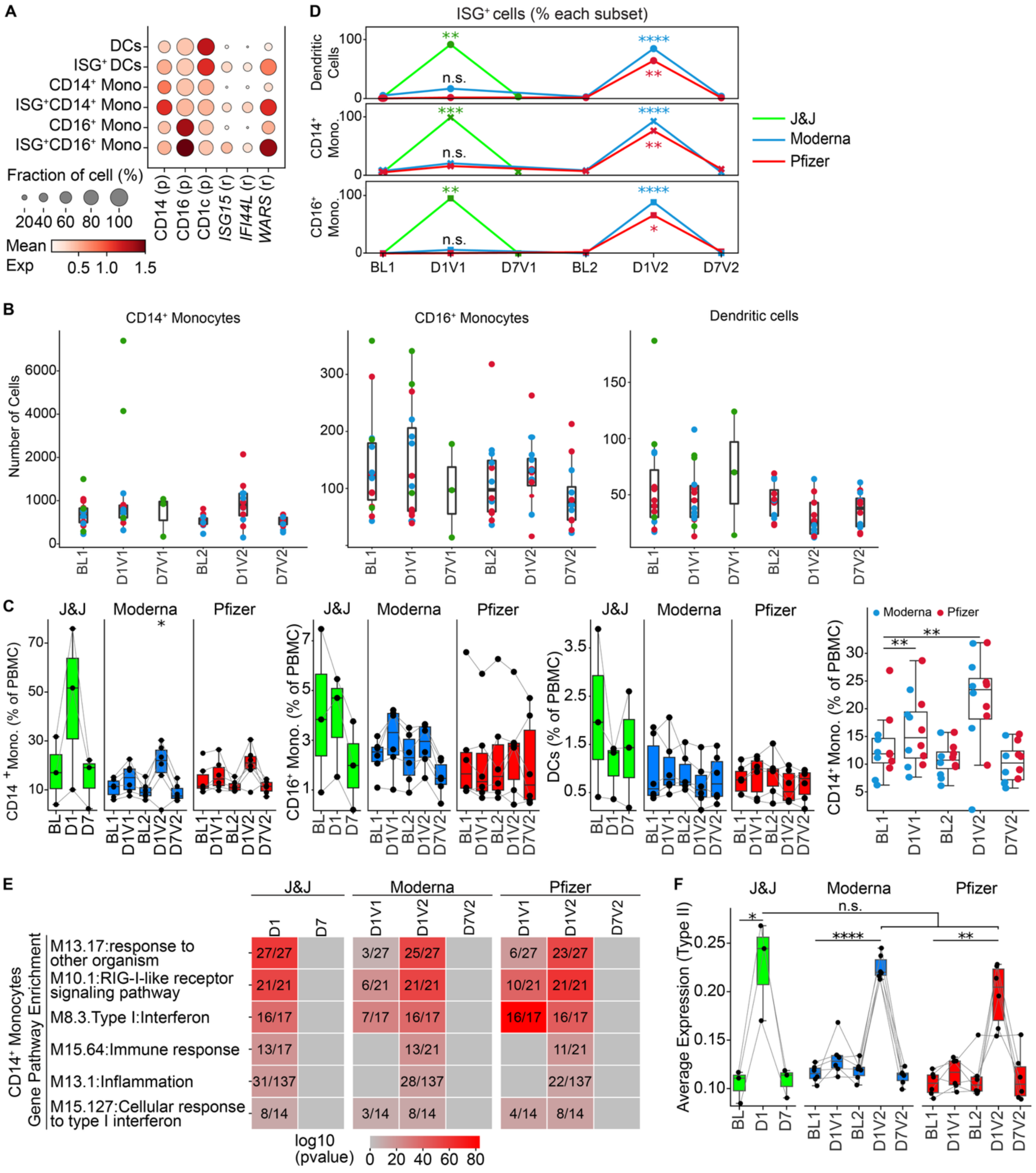

Supplementary Figure 4: Adenovirus and mRNA vaccines response in CD14⁺ monocytes.

(A) The average expression of marker genes in each myeloid and ISG subsets. (**B)** The percentage of CD14⁺ monocytes, CD16⁺ monocytes and dendritic cells (DCs) in total PBMC (left). Significant expansion of the CD14⁺ monocytes upon mRNA vaccination (right). **(C)** The number of cells is given as a box plot for each time point in myeloid cells. **(D)** The percentage of ISG subsets within each lineage for each vaccine. (**E)** The heatmap shows the top 6 enriched pathways obtained by over-representation analysis using the Blood3GenModule. The number inside indicates the

number of overlaps for the respective module. **F)** Type-II interferon expression score calculated from manually curated list (n=51). **(C)** Statistical comparisons were performed using the one-sided Wilcoxon test **(D, F)** Statistical significance between timepoints was performed using a two-tailed paired t-test; statistical significance between mRNA vaccine (Pfizer and Moderna) and adenovirus was performed using a non-paired t-test: n.s. non-significant, $*P < 0.05$, $**P < 0.01$, $***P < 0.001$, $****P < 0.0001$.

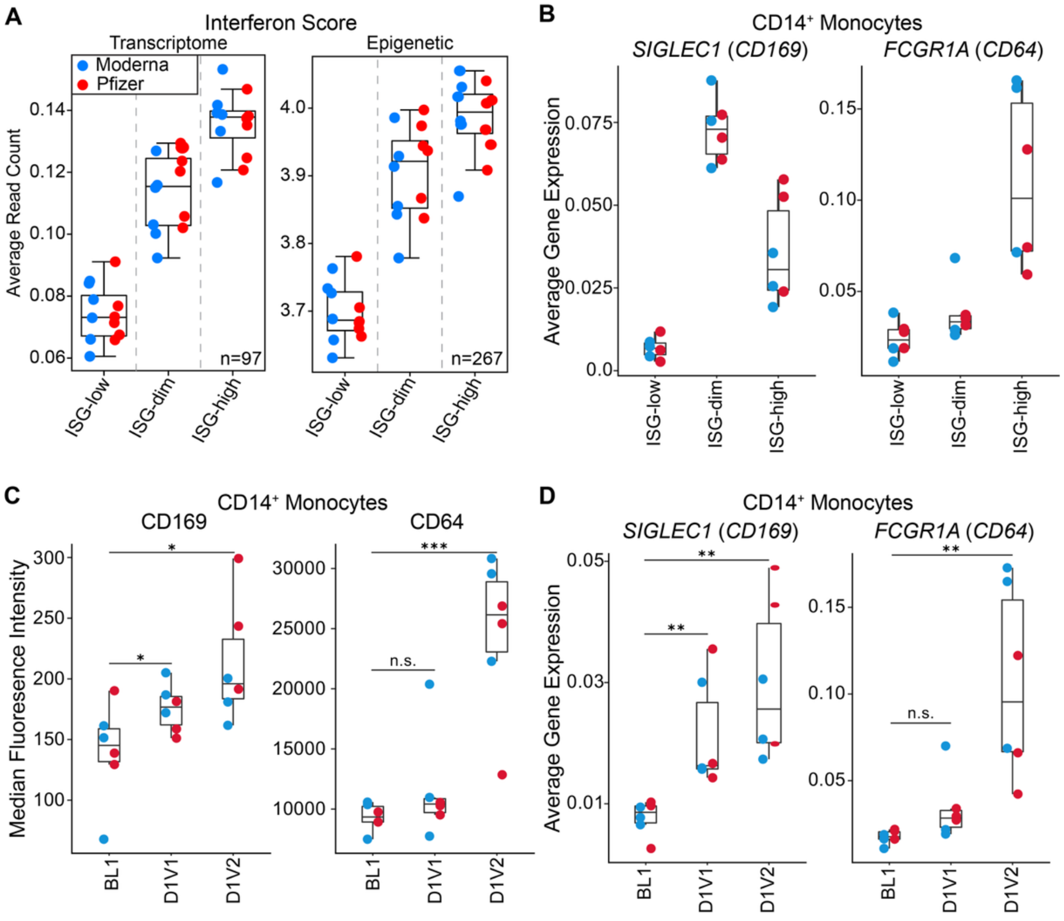

Supplementary Figure 5: The ISG transcriptome and surface protein markers to distinguish ISG-dim and ISG-high populations in CD14⁺ Monocytes.

(A) Interferon scores for cells in each ISG state. **(B)** Mean gene expression levels of *SIGLEC1 (CD169)* and *FCGR1A (CD64)* across ISG states. **(C)** Fluorescence mean intensity of CD169 (ISG-dim marker) and CD64 (ISG-high marker) measured by flow cytometry in CD14⁺ monocytes from six mRNA-vaccinated donors. **(D)** Mean gene expression levels of *SIGLEC1 (CD169)* and *FCGR1A (CD64)* across corresponding CD14⁺ monocytes from the same donors.

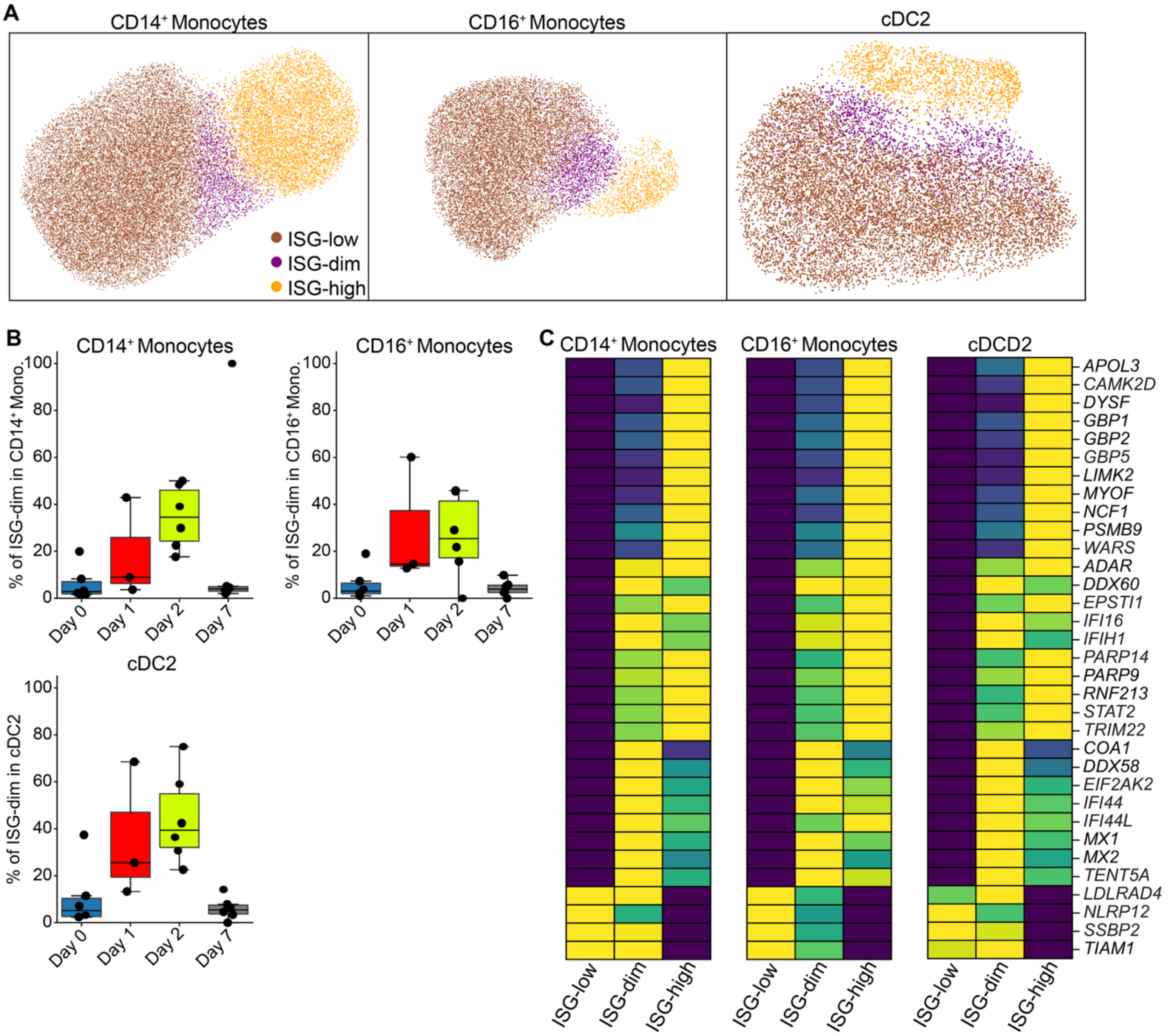

Supplementary Figure 6: Distinct ISG subset signature in publicly available data from donors vaccinated with BNT162b2.

(A) UMAP representation of ISG-low, ISG-dim, and ISG-high subsets in CD14$^+$ monocytes, CD16$^+$ monocytes and cDC2 cell types. (**B**) ISG-dim cell percentage within respective cell populations across different timepoints. **(C)** The heatmap displays the expression levels of marker genes for ISG states in CD14$^+$ monocytes, CD16$^+$ monocytes, and cDC2s.

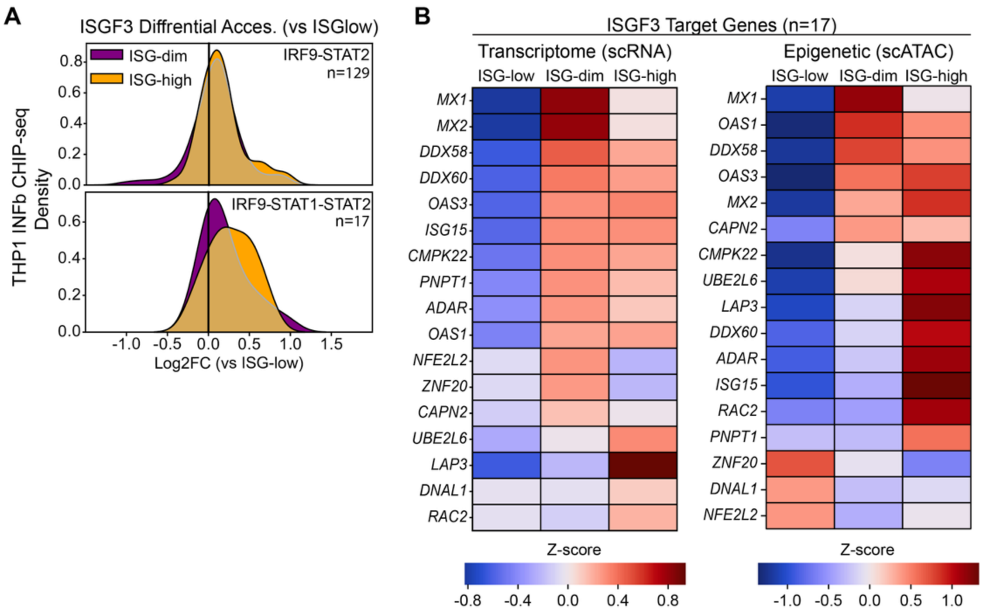

Supplementary Figure 7: ISGF3 Transcription factor epigenetic dynamic changes

A) Kernel density estimation plots show the distribution of accessibility of transcription factor binding sites in ISG-dim and ISG-high compared to the ISG-low subset. IRF9-STAT2 and IRF9-STAT1-STAT2 (ISGF3) peaks were identified by intersecting each TF motif of overlapping peaks of CHIP-seq data. **B)** The heatmap shows the expression and accessibility of the genomic region targeted by the ISGF3 complex in three ISG states.

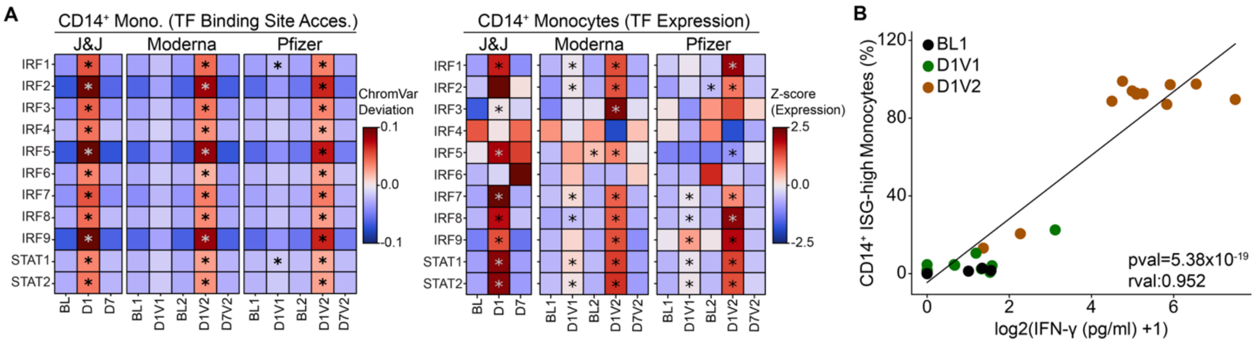

Supplementary Figure 8: IFNG cytokine levels correlates with ISG-high CD14^{+} Monocyte

(A) Longitudinal binding site activity of the IRFs and STATs calculated by ChromVAR (left); corresponding gene expression levels (right) of same TFs (right). (**B**) Pearson correlation between ISG-high state percentage in CD14^{+} monocytes and IFN-γ cytokine levels per sample. **(A)** Statistical significance between timepoints were performed using one-tailed paired t-test, $*P < 0.05$.
